# A Comparison of Colistin versus Colistin Plus Meropenem for the Treatment of Carbapenem-Resistant *Acinetobacter baumannii* in Critically Ill Patients: A Propensity Score-Matched Analysis

**DOI:** 10.3390/antibiotics9100647

**Published:** 2020-09-28

**Authors:** Wasan Katip, Suriyon Uitrakul, Peninnah Oberdorfer

**Affiliations:** 1 Department of Pharmaceutical Care, Faculty of Pharmacy, Chiang Mai University, Chiang Mai 50200, Thailand; 2 Epidemiology Research Group of Infectious Disease (ERGID), Chiang Mai University, Chiang Mai 50200, Thailand; aoberdor@med.cmu.ac.th; 3 Department of Pharmaceutical Care, School of Pharmacy, Walailak University, Nakhon Si Thammarat 80160, Thailand; Suriyon.ui@wu.ac.th; 4 Division of Infectious Diseases, Department of Pediatrics, Faculty of Medicine, Chiang Mai University, Chiang Mai 50200, Thailand

**Keywords:** critically ill patients, combination therapy, colistin, meropenem, carbapenem-resistant *Acinetobacter baumannii*

## Abstract

Carbapenem-resistant *Acinetobacter baumannii* (CRAB), an important nosocomial pathogen, occurs particularly in the intensive care unit (ICU). Thus, the aim of this study was to compare the efficacy and safety of documented treatment with colistin monotherapy versus colistin plus meropenem in critically ill patients with CRAB infections at Chiang Mai University Hospital (CMUH). We conducted a retrospective cohort study of critically ill patients with CRAB infections in an ICU from 2015 to 2017, who received colistin monotherapy versus colistin plus meropenem. After propensity score matching, an adjusted odds ratio (aOR) of a 30-day mortality rate in patients who received colistin plus meropenem was 0.43 compared to those who received colistin monotherapy (95% CI, 0.23–0.82, *p* = 0.01). aORs of clinical response and microbiological response were also higher in patients who received colistin plus meropenem (1.81, 95% CI 1.01–3.26, *p* = 0.048 and 2.08, 95% CI 1.11–3.91, *p* = 0.023, respectively). There was no significant difference in nephrotoxicity (aOR, 0.76, 95% CI, 0.43–1.36, *p* = 0.363) between colistin monotherapy and colistin plus meropenem. In conclusion, the addition of meropenem to colistin caused a reduction in 30-day mortality, higher clinical and microbiological responses, and did not increase nephrotoxicity compared to colistin monotherapy. Furthermore, 30-day mortality was significantly related with age, receiving vasopressor, having malignancy, and the APACHE II score.

## 1. Introduction

Carbapenem-resistant *Acinetobacter baumannii* (CRAB) infections are a serious problem and increasing worldwide, particularly in the intensive care unit (ICU) [1,2]. The incidence of *A. baumannii* in ICU in Europe, Eastern Mediterranean and Africa was 56.5 cases per 1000 patients. For carbapenem-resistant *A. baumannii* infections in ICUs, the incidence was 41.7 cases per 1000 patients. In ICUs, *A. baumannii* and carbapenem-resistant *A. baumannii* strains accounted for 20.9% of all hospital-acquired *A. baumannii* infections [3]. The prevalence of CRAB in other studies was 70.5–91% in Singapore, more than 90% in Vietnam and 46.7–80% in Thailand [4]. The mortality rate of *A. baumannii* infection in hospital was 35.3% and of CRAB-related infection in ICU was 60.1% [1]. Due to altered physiological characteristics related to critical illness and the care provided in an ICU, such as the common use of carbapenem and invasive procedures, critically ill patients are more susceptible to CRAB infections associated with substantially increased morbidity and mortality [2].

Colistin is an important antimicrobial agent for the treatment of CRAB infections [2,5]. Increasing rates of carbapenem resistance have led to widespread use of colistin to treat the diverse infectious diseases caused by *A. baumannii* [5,6]. However, colistin treatment failures involving *A. baumannii* have already been reported, and colistin has limitations as a therapeutic agent (i.e., a minimal post-antibiotic effect and heterogeneous colistin resistance among multidrug-resistant (MDR) isolates) [5,6,7].

Treatment strategies for CRAB infections include tigecycline, colistin, meropenem and sulbactam (as monotherapy or combination therapy) [1]. In addition to colistin, some potential therapeutic regimens have emerged. Meropenem, a carbapenem antibiotic, has a low toxicity profile and is highly resistant to serine β-lactamases produced by many MDR Gram-negative bacteria, thus playing a key role in combination therapies to reduce *A. baumannii* resistance [8]. Numerous experiments have been performed with meropenem-based therapies. A meta-analysis reported that the combination of colistin and a carbapenem (including meropenem) could efficiently suppress the development of colistin resistance and displayed a >50% synergy rate against colistin-resistant strains [9]. Moreover, the data from an in vitro pharmacokinetic/pharmacodynamic model showed that colistin combined with meropenem therapy has demonstrated a synergistic effect in CRAB strains [10].

Despite the large number of in vitro studies on CRAB, evidence that colistin should be used as monotherapy or in combination with meropenem in critically ill patients for treatment of CRAB infections is still limited. Therefore, we aimed to compare the efficacy and safety of documented treatment with intravenous colistin versus colistin plus meropenem in critically ill patients with CRAB infections.

## 2. Results

A total of 374 critically ill patients with CRAB infections were analysed. Two hundred and twenty-two patients (59%) received colistin monotherapy and 153 patients (41%) received colistin plus meropenem combination therapy. The mean age was 63.70 ± 16.76 years for patients in the colistin monotherapy group and 67.11 ± 18.13 years for patients in the colistin plus meropenem group. The main characteristics of the patients divided by the antibiotic use are presented in Table 1.

In the unmatched cohort, patients between the two groups were imbalanced in some characteristics, such as age, septic shock, mechanical ventilation during infection, Charlson score, APACHE II score, baseline serum creatinine, baseline GFR, amphotericin B, vasopressor, vancomycin, pneumonia, other sources of CRAB infection, and some comorbidities (Table 1).

**Figure 1:**
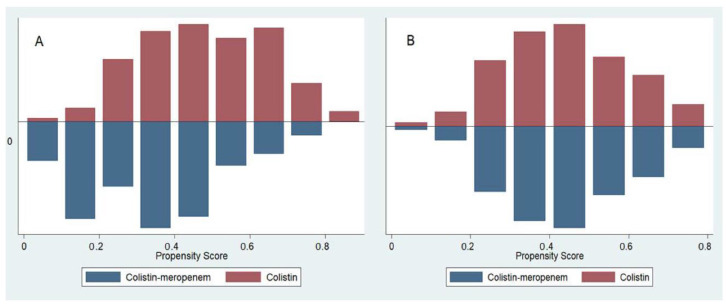
Distribution of propensity scores before matching (**A**) and after matching (**B**).

After matching patients in a 1:1 ratio using propensity scores, 248 patients were included, where 124 were assigned to the colistin monotherapy group and 124 were assigned to the colistin plus meropenem group. The characteristics of the two groups were similar with a mean ± SD propensity score of 0.44 ± 0.15. The distribution of propensity scores between groups before and after matching are shown in Figure 1. Patient characteristics between the two groups were largely similar after propensity matching (Table 1).

### 2.1. Patient Outcomes after a Propensity Scoring Match

The overall 30-day mortality rate was 50.82% (131 patients). Upon a crude comparison of the two groups, 72 (58.06%) and 59 (47.58%) patients in the colistin monotherapy and colistin plus meropenem groups, respectively, had died (*p* = 0.127). The rate of clinical response was observed (139/248, 56.04%), with clinical response being achieved in 52.42% and 59.68% of the patients in the colistin monotherapy and colistin plus meropenem groups, respectively (*p* = 0.306). The rate of microbiological response was also observed (168/248, 67.74%), with a microbiological response being achieved in 62.10% and 73.39% of the patients in the colistin monotherapy and colistin plus meropenem groups, respectively (*p* = 0.077). The rate of nephrotoxicity according to the RIFLE (Risk, Injury, Failure, Loss of kidney function, and End-stage kidney disease) criteria was 56.30% and 49.17% for the colistin monotherapy and colistin plus meropenem groups, respectively (*p* = 0.301). The analysis for crude outcomes after a propensity scoring match is shown in Table 2.

### 2.2. Unmatched Cohort Analyses

A logistic regression analysis using variables (in the statistical analysis section) for the primary outcome (30-day mortality) and secondary outcomes (i.e., clinical response, microbiological response, and nephrotoxicity) showed that the aORs for the colistin plus meropenem group were 0.39 (95% CI, 0.22 to 0.68, *p* < 0.001), 1.71 (95% CI, 1.03 to 2.83, *p* = 0.039), 1.96 (95% CI, 1.15 to 3.32, *p* = 0.013) and 0.94 (95% CI, 0.58 to 1.56, *p* = 0.835), respectively (Table 3).

### 2.3. Propensity-Matched Cohort Analyses

The results of the propensity score matching analysis using the logistic regression model were similar to those from the unmatched analysis, showing significant differences in both the primary outcome (30-day mortality) and secondary outcomes (i.e., clinical response, microbiological response, and nephrotoxicity with *p* < 0.001, *p* = 0.048, *p* = 0.023 and *p* = 0.363, respectively) (Table 3).

### 2.4. Risk Factors Associated with 30-Day Mortality

In the multivariable analysis, treatment of CRAB infections in critically ill patients with colistin plus meropenem (aOR, 0.39, 95% CI, 0.22 to 0.69) was associated with 30-day mortality compared to that from treatment with colistin monotherapy after adjusting for potential confounders (i.e., colistin plus meropenem was associated with a significantly lower risk of 30-day mortality compared to colistin monotherapy) (Table 4). We observed that the group with 30-day mortality was significantly older (mean age, 69.38 years; *p* < 0.001), had a higher proportion having received vasopressors (87.83%; *p* < 0.001), had a higher proportion of malignancy (30.69%; *p* < 0.001), and a higher median APACHE II score (median APACHE II score, 18; *p* < 0.001). (Table 4).

## 3. Discussion

This study evaluated the efficacy of colistin plus meropenem combination therapy compared with colistin monotherapy in the treatment of critically ill patients with CRAB infections. Our results indicate that 30-day mortality was reduced by the addition of meropenem to colistin. Moreover, combination treatment was associated with significantly higher clinical and microbiological responses compared to colistin monotherapy. However, nephrotoxicity was not significantly associated with combination therapy compared to colistin monotherapy. Using propensity score matching analysis with a logistic regression model, outcomes with colistin plus meropenem combination therapy were still superior to colistin monotherapy.

Colistin-based combination therapy has become an important strategy to combat CRAB [9,10,11,12]. However, whether colistin should be used as monotherapy or in combination with meropenem in critically ill patients is also unclear.

In vitro models showing synergism between colistin and meropenem support combination treatment for CRAB infections [9,10,11,12]. The potential advantages of combination treatment are improved effectiveness due to synergism and prevention of development of resistance [9]. Bian et al. evaluated the synergy of colistin in combination with meropenem against CRAB isolates. Colistin combined with meropenem exerted synergistic killing against CRAB, with an MIC of meropenem ≥32 mg/L [10]. Similar to Soudeiha et al. [12], who evaluated the effect of colistin-carbapenem combination therapy using the Checkerboard, Etest, and Time–Kill Curve Techniques, the combination therapy showed better effects compared to colistin monotherapy (*p* < 0.05). Colistin-meropenem combination therapy showed a decrease of 2.6 in the MIC of colistin. Therefore, colistin-meropenem combination therapy could be a promising antimicrobial strategy in treating CRAB infections and potentially lower the toxicity of colistin related to higher doses being used in monotherapy [12]. Moreover, a previous meta-analysis [9] revealed that the combination of colistin and a carbapenem (including meropenem) could efficiently suppress the development of colistin resistance and displayed a >50% synergy rate against colistin-resistant strains. Hence, combination therapy was put forward to alleviate the development of resistance [9]. A retrospective analytical study in patients receiving loading dose (LD) colistin monotherapy or LD colistin plus meropenem combination therapy for treatment of CRAB at Maharaj Nakorn Chiang Mai Hospital from 2013 to 2017 showed no significant difference in the clinical and microbiological cures between the two groups. Mortality rates at the end of treatment and nephrotoxicity in both groups were also not different. However, the above-mentioned study did not evaluate the clinical impact of combination therapy in critically ill patients, so the efficacy of combination therapy against multidrug-resistant *A. baumannii* in patients with severe *A. baumannii* infections and in other population might be different from the present study [13].

Our results after adjustment for confounders showed that colistin plus meropenem was associated with significantly lower 30-day mortality compared to colistin monotherapy (aOR,0.39 (95% CI, 0.22 to 0.68); *p* = 0.001). At the end of treatment, the clinical response was significantly higher in the colistin plus meropenem group compared to the colistin monotherapy group (aOR, 1.71 (95% CI, 1.03 to 2.83); *p* = 0.039). In addition, colistin plus meropenem combination therapy, compared with colistin monotherapy, had a higher microbiological response (aOR, 1.96 (95% CI, 1.15 to 3.32); *p* = 0.013), but no difference in nephrotoxicity (aOR, 0.94 (95% CI, 0. 58 to 1.56); *p* = 0.835).

The findings of our study were similar to those of a previous study by Shields et al. [11], who compared the use of colistin-based antibiotic combinations against XDR *Acinetobacter* in vitro and demonstrated positive interactions for the combination of colistin and a carbapenem. Subsequently, 80% (4/5) of transplant patients were treated successfully with regimens consisting of the combination of colistin and a carbapenem. However, the number of subjects in this study was small and without a comparison group [11].

A recent randomised trial [14] compared colistin plus meropenem versus colistin monotherapy in carbapenem-resistant gram-negative bacterial infections. The study demonstrated that the addition of meropenem to colistin did not improve clinical failure in carbapenem-resistant gram-negative bacterial infections. However, the study did not focus on evaluating the clinical impact of combination therapy in critically ill patients and did not measure drug concentrations [14]. Thus, the advantages of combination treatment may have been different among studies.

Our findings showed that the maximal clinical benefit of combination therapy was observed in our patients with critical illness, which is consistent with data from a previous study [15,16]. A retrospective study comparing colistin monotherapy with colistin plus a carbapenem combination therapy on 160 cases of CRAB pneumonia showed no significant difference in 14-day mortality between the combination and monotherapy groups (24.1% vs. 20.8%, *p* = 0.616). However, after adjusting for disease severity according to APACHE II score, the 14-day mortality was significantly lower in the combination group compared to the monotherapy group among patients with APACHE II scores of 25–29 points (9.1% vs. 53.8%, *p* = 0.020) [15]. In a previous retrospective study that evaluated data from 71 patients with CRAB bacteraemia treated with colistin monotherapy and colistin plus meropenem combination therapy, mortality tended to be higher in the monotherapy group, but the difference was not statistically significant (47.5% vs. 25.8%). However, combination therapy was significantly more efficacious in patients with a high Pitt bacteremia score (66.7% vs. 27.8%, *p* = 0.036) [16].

In our study, crude analysis before adjustment of confounding factors indicated that 30-day mortality rate was not significant different between patients in the colistin monotherapy and colistin plus meropenem groups (58.06% and 47.58%, respectively, *p* = 0.127). This insignificance could be caused by differences in baseline and treatment characteristics although propensity score matching were performed. After adjustment of the confounders using multivariate analysis, colistin-meropenem combination therapy showed significantly promising results. The proposed mechanism of this synergistic effect is combined effect of the two molecules on bacterial cells. Colistin acts against the outer bacterial membrane, changing its permeability [17], which in turn allows meropenem to enter the bacteria in higher concentrations [18]. Higher concentrations of meropenem in the periplasmic space could reduce the bacterial resistance mechanisms, thereby rendering meropenem active against resistant bacteria [19].

However, a secondary analysis of the Affirmative Integrated Energy Design Action (AIDA) trial [20], which evaluated the synergistic interaction between colistin and meropenem for each isolate in vitro, found that patients infected with strains for which the drugs were synergistic did not have better clinical outcomes, and patients with strains for which the drugs were antagonistic did not have worse outcomes. In fact, all outcomes were worse in the synergism group compared to the antagonism group, possibly reflecting residual confounding. So, the AIDA trial failed to prove overall clinical benefits [20].

We performed a multivariable regression analysis to elucidate factors that were independently associated with 30-day mortality in our study cohort. In addition to the stepwise inclusion of variables that were found to be different (*p* ≤ 0.25) between patients who were survivors and those who were non-survivors, we also included variables that were different between the treatment groups to account for potential selection bias. After adjusting for potential confounders in baseline characteristics, we found that the treatment of CRAB infections with colistin plus meropenem was associated with approximately 0.4 times the risk of 30-day mortality compared to treatment with colistin monotherapy. In other words, colistin plus meropenem was associated with a significantly lower rate of 30-day mortality than colistin monotherapy in our adjusted analysis. A higher proportion of patients having received vasopressors, higher proportion of malignancy, higher APACHE II scores and older age were also found to independently increase the risk of 30-day mortality. The incidence of nephrotoxicity was similar across all treatment groups (56.30 vs. 49.17, *p* = 0.301). The rates of nephrotoxicity observed in our study were comparable to the rates reported in previous studies based on the RIFLE criteria, which ranged from 20% to 69%, whereas higher daily doses were found to cause renal toxicity, which is dose-dependent [21,22,23].

However, some limitations in this study should be noted. First, we observed significant differences in baseline characteristics between the treatment groups, although this difference was also found in most retrospective studies and was difficult to make similar in both groups, which may have resulted in confounding. However, a propensity score-matching method was used to adjust for known baseline characteristics. Moreover, in an attempt to address the most important confounders, we conducted a multivariable regression analysis, ensuring that statistically significant confounders with clinical plausibility were maintained in our final multivariable model. Second, as the study was conducted at a single centre, the patient characteristics and distribution of genetic mechanisms of resistance could differ according to local epidemiology, which might have influenced the effect of combination therapy.

## 4. Materials and Methods

A retrospective cohort study was conducted at Chiang Mai University Hospital (CMUH), a tertiary care teaching hospital in Chiang Mai, Thailand, from January 2015 to August 2017. This study was approved by the ethics committee on human research of the Faculty of Medicine, Chiang Mai University of a waiver of informed consent for retrospective data collection under the condition of anonymously stored data collected. All methods were performed in accordance with the relevant guidelines and regulations. The patients had monomicrobial infection due to carbapenem-resistant and colistin-sensitive *A. baumannii*. Patients with CRAB infections were identified using a microbiology database source and medical chart records. The criteria used to identify and classify infections were those of the US Centers for Disease Control and Prevention (CDC) [24] and according to the evaluations of infectious disease (ID) physicians. Patients were included if they were critically ill, ≥18 years of age and admitted to the intensive care unit (ICU), who received colistin for more than 2 days to treat a documented CRAB infection and received only one course of colistin treatment. Only patients who did not receive any other drug with potential activity against *A. baumannii* were included. Patients with CRAB cultures assessed to be colonisers or contaminants or who had incomplete records were excluded. The patients were divided in two groups: colistin monotherapy versus colistin plus meropenem. The inclusion criterion for patients in the combination therapy group was the administration of meropenem for more than 48 h in combination with colistin. Colistin was administered by the intravenous (i.v.) route with a loading dose of 300 mg (9 million units of colistimethate sodium) of colistin base activity (CBA), followed by 150 mg CBA every 12 h (corrected according to renal function). Meropenem was administered at a dose of 1000 mg i.v. over 1 h every 8 h.

### 4.1. Outcomes Measurement

Efficacy was assessed based on 30-day mortality, clinical responses, and bacteriological responses to the therapy. Primary outcome in this study was 30-day mortality, defined as death for any cause occurring within 30 days of initial treatment. The secondary outcomes of interest included clinical response to treatment, which was assessed by resolution or partial resolution of presenting symptoms and signs of CRAB infection at the end of colistin treatment. Clinical failure was defined as failure to meet all criteria for clinical response during colistin treatment. Other secondary outcomes of interest included microbiological response at the end of therapy, defined as a follow-up of two consecutive CRAB-negative cultures of clinical samples obtained from the site of infection after the initial positive culture, whereas microbiological failure was defined as persistence of CRAB in the subsequent specimen cultures. Safety data were assessed as clinical signs and symptoms and via laboratory findings. Nephrotoxicity was defined according to the RIFLE criteria [25]. Evidence of nephrotoxicity from colistin was obtained from the review of physicians’ notes. Nephrotoxicity was counted if patients developed any grade of renal failure based on the RIFLE criteria.

### 4.2. Antimicrobial Susceptibility Testing

Identification of all causative microorganisms was performed using routine microbiological methods. Susceptibility testing was performed by both the disk diffusion method and an automated bacterial identification and susceptibility testing system (VITEK 2 system, bioMérieux, Marcy I ’Etoile, France). Antimicrobial susceptibility was evaluated according to the Clinical and Laboratory Standards Institute (CLSI) protocol [26]. Susceptibility of *A. baumannii* to antibiotics was assessed by the VITEK 2 system, and susceptibility to colistin was assessed by broth microdilution, with resistance defined as having a colistin minimum inhibitory concentration (MIC) breakpoint > 2 mg/L. The VITEK 2 system is a fully automated system utilising a fluorogenic methodology for organism identification and a turbidimetric method for susceptibility testing [27]. CRAB was defined as *A. baumannii* resistant to carbapenems, but sensitive to colistin.

### 4.3. Statistical Analysis

Data analysis was performed using Stata software, version 14 (Stata Corp, College Station, TX, USA). The two treatment groups were compared on the basis of the duration of colistin treatment.

Descriptive statistics were used to describe the data, including percentage, frequency, average, and standard deviation to describe the general characteristics and basic information of the patients, for comparing the main outcome differences of the study. The average comparison case of sample basic data was performed by using Fisher’s exact test. The averages of other statistical methods were obtained by an independent T-test when data were distributed normally and by a Mann–Whitney U test when data were non-normally distributed, with a set significance level of <0.05.

Due to imbalances in the baseline characteristics of the treatment groups, propensity score matching was performed to reduce potential biases. We calculated a propensity score using multivariable logistic regression. The variables included in the propensity score calculation were age, hypertension, cardiovascular disease, diabetes mellitus, chronic kidney disease, liver disease, septic shock, mechanical ventilation during infection, Charlson score, APACHE II score, baseline serum creatinine, baseline glomerular filtration rate (GFR), amphotericin B, vasopressor, vancomycin, pneumonia, other sources of CRAB infection and baseline covariates with an inclusion criterion of *p* < 0.25.

Fisher’s exact test was used after matching the propensity score to compare differences in rates of 30-day mortality, clinical response, microbiological response and nephrotoxicity based on colistin monotherapy and colistin plus meropenem combination therapy.

Furthermore, a logistic regression model was used to estimate the odds ratio (OR) between colistin monotherapy, colistin plus meropenem and outcomes for both the primary outcome (30-day mortality) and secondary outcomes (i.e., clinical response, microbiological response, and nephrotoxicity). The variables likely to influence the outcomes were age, gender, comorbidities, courses of colistin therapy, septic shock, Charlson score, APACHE II score, chronic liver disease, baseline serum creatinine, malignancy, mechanical ventilation during infection, total colistin dose, vasopressor, vancomycin, aminoglycosides, amphotericin B, cardiovascular disease, diabetes mellitus, chronic kidney disease, and other sources of CRAB infections. First, univariate analyses were performed to evaluate the predictive effect of each factor alone. Then, in addition to treatment, any factor whose univariate test result had a *p* < 0.25 was included in a full multiple logistic model. Finally, the full model was reduced one factor at a time in such a way that all factors remaining in the model were statistically significant at a 5% significance level, except colistin plus meropenem combination therapy remained in the model (regardless of its *p* value).

To identify independent predictors of 30-day mortality in all critically ill patients, covariates with *p* ≤ 0.25 in the univariate mortality analysis and other variables that demonstrated a trend toward association with outcomes were entered into the logistic regression multivariable model in a backward stepwise elimination. Finally, the full model was reduced one factor at a time in such a way that all factors remaining in the model were statistically significant at a 5% significance level, regardless of their *p* values. The results of the logistic regression were expressed as the adjusted odds ratio (aOR) and 95% confidence interval (CI).

## 5. Conclusions

In critically ill patients with CRAB infections, the combined treatment with colistin plus meropenem was associated with significantly lower mortality when compared with colistin monotherapy. Moreover, combination therapy was associated with significantly higher clinical and microbiological responses compared to monotherapy with colistin. However, there was no difference in nephrotoxicity between colistin plus meropenem and colistin monotherapy. Therefore, these results suggest that the combination of colistin and meropenem might be a promising therapy against CRAB infections in critically ill patients. Furthermore, the risk factors for 30-day mortality was significantly related with old age, vasopressor use, malignancy diagnosis, and a high median APACHE II score.

## Figures and Tables

**Table 1:** Demographic and clinical characteristics of patients who received colistin monotherapy compared to colistin-meropenem combination therapy.

Characteristic	Unmatched Cohort			Propensity-Matched Cohort		
	Colistin Monotherapy (*n* = 221)	Colistin-Meropenem (*n* = 153)	*p* Value	Colistin Monotherapy (*n* = 124)	Colistin-Meropenem (*n* = 124)	*p* Value
Sex, *n* (%)						
Male	79 (35.75)	64 (41.83)	0.236	41 (33.06)	49 (39.52)	0.355
Female	142 (64.25)	89 (58.17)		83 (66.94)	75 (60.48)	
Age, mean ± SD (year)	63.70 ± 16.76	67.11 ± 18.13	0.061	65.75 ± 16.69	67.5 ± 18.02	0.431
Duration of treatment, months, median (IQR)	10 (7–14)	10 (6–14)	0.648	10 (6.5–14)	10 (6.5–14)	0.979
Comorbidities *, *n* (%)	185 (83.71)	142 (92.81)	0.011	110 (88.71)	114 (91.94)	0.520
• Hypertension	94 (42.53)	79(51.63)	0.092	63 (50.81)	60 (48.39)	0.800
• Cardiovascular disease	66 (29.86)	60 (39.22)	0.075	40 (32.26)	45 (36.29)	0.593
• Diabetes mellitus	41 (18.55)	44 (28.76)	0.024	28 (22.58)	29 (23.39)	1.000
• Chronic kidney disease	46 (20.81)	45 (29.41)	0.066	36 (29.03)	36 (29.03)	1.000
• Chronic obstructive pulmonary disease	38 (17.19)	30 (19.61)	0.587	19 (15.32)	26 (20.97)	0.323
• Malignancy	49 (22.17)	29 (19.08)	0.518	21 (16.94)	23 (18.55)	0.868
• Chronic liver disease	11 (4.98)	13 (8.50)	0.200	5 (4.03)	10 (8.06)	0.287
Septic shock	138 (62.44)	129 (84.31)	0.001	100 (80.65)	101 (81.45)	1.000
Mechanical ventilation	191 (86.43)	143 (93.46)	0.040	115 (92.74)	114 (91.94)	1.000
Charlson score, median (IQR)	2 (0–4)	2 (1–4)	0.198	2 (1–3.5)	2 (1–4)	0.386
Length of hospital stay, median (IQR) (day)	31.5 (22-48)	39 (25–56)	0.094	32 (22–49)	38 (23-54)	0.154
APACHE II score **, mean ± SD	16.28 ± 4.65	18.07 ± 4.97	0.001	17.08 ± 4.47	17.32 ± 4.53	0.672
Baseline SCr, mg/dL, median (IQR)	0.8 (0.5–1.4)	1.1 (0.6–1.8)	0.003	0.8 (0.5–1.3)	1.1 (0.6–1.75)	0.037
Baseline GFR, mL/min, median (IQR)	64.93 (19.61–103.3)	41.85 (14.24–79.74)	0.013	58.64 (12.71–93.73)	45.07 (17.33–81.46)	0.306
Total colistin dose, g, median (IQR)	1.95 (1.25–3.00)	1.73 (1.03–2.85)	0.025	1.75 (1.20–3.00)	1.80 (1.05–2.85)	0.460
Type of nephrotoxic medications ^#^, *n* (%)						
• Aminoglycosides	6 (2.71)	1 (0.65)	0.248	5 (4.03)	1 (0.81)	0.213
• Diuretics	179 (81.00)	126 (82.35)	0.787	108 (87.10)	104 (83.87)	0.589
• Amphotericin B	12 (5.43)	15 (9.80)	0.154	8 (6.45)	11 (8.87)	0.634
• Vasopressors	143 (64.71)	131 (85.62)	0.001	101 (81.45)	103 (83.06)	0.868
• Vancomycin	127 (57.47)	112 (73.20)	0.002	89 (71.77)	88 (70.97)	1.000
• Site of CRAB infection						
• Pneumonia	187 (84.62)	139 (90.85)	0.085	109 (87.90)	111 (89.52)	0.841
• Bacteraemia	1 (0.45)	2 (1.31)	0.570	0 (0.00)	2 (1.61)	0.498
• UTI	17 (7.69)	11 (7.19)	1.000	7 (5.65)	10 (8.06)	0.617
• Other *	25 (11.31)	12 (7.84)	0.295	11 (8.87)	10 (8.06)	1.000
Propensity score, mean + SD	0.34 ± 0.18	0.49 ± 0.17	0.001	0.44 ± 0.15	0.44 ± 0.15	0.761

SCr, serum creatinine; GFR, glomerular filtration rate; SD, standard deviation; UTI, urinary tract infection; *, intercostal drainage and surgical site infection; IQR, interquartile range; **, APACHE II, Acute Physiology And Chronic Health Evaluation II; ^#^, each patient could have more than one drug.

**Table 2:** Primary and secondary outcomes for the propensity-matched patients who received colistin monotherapy compared to colistin-meropenem combination therapy.

Outcome	No. of Patients (%) with Each Outcome with Indicated Treatment		*p* Value
	Colistin Monotherapy (*n* = 124)	Colistin-Meropenem (*n* = 124)	
**Primary outcome**			
30-day mortality rate	72 (58.06)	59 (47.58)	0.127
**Secondary outcomes**			
Clinical response	65 (52.42)	74 (59.68)	0.306
Microbiological response	77 (62.10)	91 (73.39)	0.077
Nephrotoxicity	67 (56.30)	59 (49.17)	0.301

**Table 3:** Association between colistin-meropenem combination therapy and outcomes for critically ill patients.

	Logistic Regression Analysis		Propensity Score Matched Logistic Regression Analysis	
Variable	aOR (95% CI)	*p*-Value	aOR (95% CI)	*p*-Value
**Efficacy** **Primary outcome**				
30 days mortality	0.39 (0.22–0.68)	0.001	0.43 (0.23–0.82)	0.010
**Secondary outcomes**				
Clinical response	1.71 (1.03–2.83)	0.039	1.81 (1.01–3.26)	0.048
Microbiological response	1.96 (1.15–3.32)	0.013	2.08 (1.11–3.91)	0.023
**Safety**				
Nephrotoxicity	0.94 (0.58–1.56)	0.835	0.76 (0.43–1.36)	0.363

CI, confidence interval; aOR, adjusted odd ratio.

**Table 4:** Multivariable logistic regression model for significant predictors of risk factors for 30-day mortality among all patients with CRAB infections.

Variable ^a^	Survivors (*n* = 185)	Non-Survivors (*n* = 189)	aOR (95% CI) ^b^	*p* Value
**Colistin plus meropenem**	78 (42.16)	75 (39.68)	0.39 (0.22–0.68)	0.001
**Age**	60.72 ± 18.66	69.38 ± 14.91	1.04 (1.02–1.05)	0.001
**Vasopressor**	108 (58.38)	166 (87.83)	5.56 (2.94–10.52)	0.001
**Malignancy**	20 (10.81)	58 (30.69)	3.65 (1.90–7.04)	0.001
**APACHE II score**	15 (12–19)	18 (14–21)	1.17 (1.09–1.26)	0.001

^a^ Other factors that were included in the multivariate regression model, but that were not significant, included pneumonia, diabetes mellitus, mechanical ventilation and comorbidities. Factors that were evaluated but did not remain in the stepwise backward regression model included baseline serum creatinine, gender, Charlson score, septic shock, vancomycin, hypertension, cardiovascular disease, chronic kidney disease, total colistin dose, duration of treatment and other site of CRAB infection. ^b^ The model obtained had an area under the receiver operating characteristic curve (AUC) of 0.822.
